# Comparative Analysis of the Cytotoxic Effects of Modified Triple Antibiotic Hydrogel: Insights From Experimental Models

**DOI:** 10.7759/cureus.62662

**Published:** 2024-06-19

**Authors:** Annie Sylvea Valan, Jogikalmat Krithikadatta, Rajalakshmanan Eswaramoorthy, Ajay Guru

**Affiliations:** 1 Department of Conservative Dentistry and Endodontics, Saveetha Dental College and Hospitals, Saveetha Institute of Medical and Technical Sciences, Saveetha University, Chennai, IND; 2 Department of Cariology, Saveetha Dental College and Hospitals, Saveetha Institute of Medical and Technical Sciences, Saveetha University, Chennai, IND; 3 Department of Biochemistry, Center of Molecular Medicine and Diagnostics, Saveetha Dental College and Hospitals, Saveetha Institute of Medical and Technical Sciences, Saveetha University, Chennai, IND

**Keywords:** zebrafish, mtt assay, modified triple antibiotic paste, intracanal medicaments, hydrogel, cytotoxicity

## Abstract

Introduction: The effectiveness of intracanal medicaments (ICMs) in root canal therapy is critical for successful dental treatments, yet their cytotoxic effects pose significant challenges. This research uses zebrafish embryos and dental pulp stem cells (DPSCs) to identify the optimal concentration that balances antibacterial efficacy with minimal toxicity.

Aim: This study aims to address the need for effective ICMs in dentistry by formulating and assessing the embryotoxicity and cytocompatibility of a novel carrageenan-based modified triple antibiotic paste (MTAP) hydrogel at different concentrations (1, 5, and 10 mg/mL) using a zebrafish model and cell culture assay.

Materials and methods: The hydrogel was formulated by combining antibiotic solutions (ciprofloxacin, metronidazole, and amoxicillin) with carrageenan and xanthan gum. Zebrafish embryos were exposed to varying concentrations of MTAP hydrogel, chlorhexidine (CHX), calcium hydroxide (CaOH_2_), and plain carrageenan to assess developmental toxicity, survival rate, heart rate, hatching rate, and macrophage migration. The cytotoxicity against DPSCs was examined within a timeframe of 6, 24, and 72 hours with the use of the 3-[4,5-dimethylthiazol-2-yl]-2,5 diphenyltetrazolium bromide (MTT) assay.

Results: The analysis revealed developmental toxicity with malformations observed at higher concentrations of MTAP hydrogel, CaOH_2_, and CHX medicaments, indicating potential toxicity. Significant impacts on survival, heart rate, and hatching rate were noted in the CaOH_2_ and CHX groups, as well as at higher MTAP hydrogel concentrations, emphasizing the importance of dosage considerations. The neutral red assay confirmed toxicity, with macrophage migration observed in CaOH_2_, CHX, and higher MTAP hydrogel concentrations. Lower concentrations, particularly at 1 mg/mL, showed no adverse effects on zebrafish embryos and larvae. These findings align with cell viability investigations, which demonstrated that higher antibiotic concentrations resulted in decreased cell proliferation and viability over time. Conversely, at a lower concentration of 1 mg/mL, cell proliferation notably increased after 72 hours. Plain MTAP and CHX exhibited the highest toxicity levels in the MTT assay.

Conclusion: The study concludes that while higher concentrations of MTAP hydrogel exhibit toxic effects, the hydrogel at 1 mg/mL demonstrates no adverse impact on zebrafish embryos, larvae, and DPSCs. These findings underscore the necessity of optimizing ICM concentrations to balance antibacterial efficacy and minimal cytotoxicity.

## Introduction

Odontogenic infections pertain to infections originating from the anatomical components of the teeth. Comparable to other infections, these infections involve a diverse array of microorganisms, necessitating the utilization of multiple antibiotic agents to address the specific species accountable for the pathological condition effectively [1]. Efficient decontamination is imperative for effectively managing odontogenic lesions attributed to endodontic origins. While endodontic instrumentation and irrigation serve as the primary modalities for achieving decontamination and disinfection, specific circumstances warrant the application of medicaments within the canal due to the prevalence of resistant microorganisms. The significance of intracanal medicaments (ICMs) is of paramount importance, especially in cases involving persistent lesions. ICMs are substances specifically formulated to create a sterile environment within root canals, targeting and eliminating pathogenic microorganisms [2].

Among these, calcium hydroxide (CaOH_2_) is one of the most widely used ICMs, as reported in the literature, but it has demonstrated limited antibacterial potency against *Enterococcus faecalis* [3]. Chlorhexidine (CHX), characterized as a cationic biguanide, is another frequently employed ICM. However, its effectiveness improves when combined with other medicaments rather than when used alone. To address these issues, alternative medicaments, such as triple antibiotic paste (TAP), which is a combination of ciprofloxacin, metronidazole, and minocycline, are advocated [4]. Metronidazole, classified as a nitroimidazole compound, demonstrates particular toxicity against anaerobes and is an antimicrobial agent targeting protozoa and anaerobic bacteria. Minocycline, a bacteriostatic agent, exhibits activity against both gram-positive and gram-negative bacteria, and it also induces an increase in interleukin-10 levels, an inflammatory cytokine. Furthermore, ciprofloxacin, a synthetic fluoroquinolone, possesses rapid bactericidal effects and displays strong antimicrobial activity against gram-negative bacteria [2]. This combination exhibits potent efficacy against an extensive spectrum of bacteria, encompassing both obligate and facultative types, irrespective of their gram-positive or gram-negative nature, thus facilitating the healing process [5]. Nonetheless, utilizing TAP also presents certain limitations, encompassing the potential for discoloration of teeth, alterations to dentin's structural and functional attributes, and its cytotoxic impact at elevated concentrations. Consequently, to alleviate the potential of discoloration, an alternative approach involves the application of modified TAP (MTAP), where minocycline is substituted in TAP formulations with amoxicillin, clindamycin, or cefaclor. Among these, amoxicillin emerges as the antibiotic of choice for addressing odontogenic infections based on the conclusions drawn by Kuriyama et al. [6] and Stein et al. [7]. Therefore, this study uses amoxicillin as a substitute for minocycline in preparing the modified triple antibiotic hydrogel. Thomson and Kahler conducted a previous study evaluating revascularization with an amoxicillin-containing TAP, which yielded a successful treatment outcome for an infected immature premolar with no observed discoloration [8].

The cytocompatibility of an ICM is a crucial criterion for its use, ensuring that it does not cause adverse effects on the surrounding tissues and supports the healing process within the root canal system. According to research conducted by Ruparel et al., elevated concentrations of antibiotics negatively impact the survival of stem cells of the apical papilla (SCAP) [9]. Research by Althumairy et al. demonstrated that while antibiotic pastes at concentrations around 1,000 mg/mL are lethal to SCAP survival, this harmful effect can be significantly avoided using these medicaments at 1 mg/mL [10]. These investigations highlight the importance of using ICMs at concentrations that possess bactericidal properties while causing minimal influence on the viability of stem cells.

Various models have been employed to evaluate the cytotoxicity of materials, including cell culture assays and the zebrafish larvae model. Zebrafish are widely utilized model organisms in biomedical research due to their genetic similarities to humans, transparent embryos facilitating easy observation, and rapid reproductive cycle [11]. Their susceptibility to toxins and rapid chemical absorption and distribution have also rendered them valuable in toxicology studies [12]. These characteristics make them ideal for assessing cytotoxicity, which is why they were employed in this study. Moreover, no previous studies have assessed the cytotoxic effect of ICMs using the zebrafish model, making this research novel and unique.

The rationale of this study was to evaluate the toxicity of a novel modified triple antibiotic hydrogel, which uses carrageenan, a marine biopolymer, as the carrier agent. Additionally, the study focused on using low concentrations of antibiotics, which are considered conducive to the periapical tissues. By utilizing cell culture assays and the zebrafish larvae model, the study seeks to ensure that the medicament does not adversely affect cellular viability or organismal development, thereby validating its potential for safe clinical application in endodontic treatments. A preceding investigation focused on the characterization of MTAP hydrogel, followed by an evaluation of its antibacterial efficacy, demonstrating its superior antimicrobial properties [13].

The aim of this study was to compare the embryotoxicity and cytocompatibility of carrageenan-based modified triple antibiotic hydrogel at various concentrations using a zebrafish model and cell culture assay, respectively.

## Materials and methods

Formulation of the hydrogel

The research was conducted at Saveetha Dental College and Hospitals, Saveetha Institute of Medical and Technical Sciences, Saveetha University, Chennai, India. It was conducted in March 2023 with the approval of the Institutional Scientific Review Board under the reference number SRB/SDC/ENDO-2103/23/079.

In this investigation, low concentrations of MTAP solution (1, 5, and 10 mg/mL) were prepared for the formulation of hydrogels using carrageenan and xanthan gum, employing a methodology outlined in prior studies [13]. The process began by combining equal quantities of amoxicillin, ciprofloxacin, and metronidazole in sterile water to prepare the MTAP solutions. This mixture gradually dissolved to ensure complete and uniform mixing of the antibiotics. The dissolution occurred in a sterile environment with continuous agitation to facilitate proper mixing. Following the dissolution of the antibiotics, 0.5% carrageenan and xanthan gum powder were introduced into each MTAP solution. These additives were carefully incorporated into the solutions while maintaining vigorous and consistent stirring. This meticulous approach aimed to form hydrogels at 1, 5, and 10 mg/mL concentrations by uniform dispersion of the carrageenan and xanthan gum throughout the MTAP solutions.

Origin and management of zebrafish

Mature zebrafish (wild type-AB strain) were obtained from a nearby aquarium supplier (NSK Aquarium, Kolathur, Tamil Nadu, India). Upon arrival, male and female zebrafish were housed separately in a controlled environment. They were accommodated in a 10-L glass tank, with the water temperature maintained at 28.5°C and a light/dark cycle of 14/10 hours. The zebrafish were fed live brine shrimp (*Artemia salina*), administered thrice daily. After a month of acclimation, the fish were used for breeding, and the embryos were collected for further research. When examined under a microscope, only fertilized embryos were retained, while unfertilized ones were discarded. After fertilization, the embryos were placed on a six-well plate and cultured in an E3 medium [14].

Developmental toxicity in embryos

Zebrafish embryos are widely used in developmental toxicity analysis due to their transparent body and rapid development, which allows for easy observation of developmental defects and abnormalities in embryos [15]. In this study, the evaluation of cytotoxicity included the use of various compounds, including CaOH_2_, 2% CHX as an ICM, and MTAP hydrogel at 1 mg, 5 mg, and 10 mg/mL, alongside plain carrageenan and a control group. These medicaments were administered to zebrafish subjects for analysis. Zebrafish were exposed to the solutions containing these substances for a specified duration of 24 hours. Subsequently, the developmental stages of the embryos were observed under an inverted microscope to identify and examine potential malformations [16].

Heart rate in zebrafish embryos

Heart rate analysis in zebrafish embryos is a technique used to measure the heart rate of developing zebrafish embryos. Zebrafish embryos are widely used as model organisms for studying heart development and function due to their transparent bodies, which allow for easy visualization of the developing heart. Zebrafish embryos demonstrate optimal conditions for capturing the complete heartbeat frequency at 48 hours postfertilization (hpf). To conduct heart rate analysis in zebrafish embryos, the embryos are typically immobilized in a small amount of agarose and then placed under a microscope with a high-speed camera. The camera records the heart's movement, and specialized software is used to analyze the images and measure the heart rate [17]. 

Hatching rate in zebrafish embryos

Hatching rate analysis in zebrafish embryos is a technique used to measure the proportion of embryos that successfully hatch from their chorions, which are the protective outer shells surrounding the developing embryos. Zebrafish embryos typically hatch at around 48-72 hpf, and a decrease in hatching rate can indicate developmental abnormalities or exposure to toxins or other stressors. To conduct hatching rate analysis in zebrafish embryos, the embryos are typically placed in a solution containing a chemical that dissolves the chorion, allowing the embryos to hatch. The number of hatched embryos is then counted and compared to the total number of embryos used in the experiment to calculate the hatching rate. The count of hatched larvae was recorded for each treatment group, and the hatching rate (%) was calculated using the formula: (number of hatched larvae / total number of exposed larvae) × 100 [18].

Survival rate in zebrafish embryos

The evaluation of survival rates in zebrafish embryos is a widely employed method to analyze the toxicity of various chemicals and environmental factors on the development and viability of these embryos. Zebrafish embryos are frequently used due to their translucent bodies, small size, and rapid development. To conduct survival rate analysis in zebrafish embryos, the embryos are typically exposed to 1, 5, and 10 mg/mL of modified triple antibiotic hydrogel, plain carrageenan, a control group, and gold standard medicaments such as CaOH_2_ and 2% CHX. The number of surviving embryos was recorded daily, and any deceased embryos were promptly removed. The embryos are then monitored over a period of time to determine the percentage that survive at each concentration [19].

Neutral red assay in zebrafish embryos

The neutral red assay is a widely used technique for assessing macrophage function in zebrafish larvae. Live larvae are typically incubated with a neutral red dye solution for a short duration to conduct the neutral red assay in zebrafish larvae. The dye is taken up by the macrophages and accumulates in their lysosomes, which are small organelles within the cells that break down and digest foreign substances. After incubation with the dye, the larvae are washed to remove excess dye and then anesthetized and imaged under a microscope. The macrophages appear as red spots within the larvae, and specialized software can be used to count the number of macrophages in each larva [20].

Culture of DPSCs

To culture dental pulp stem cells (DPSCs), Eagle's minimum essential medium F12 was utilized with supplementation of 15% (vol/vol) heat-inactivated fetal bovine serum, 2 mM L-glutamine, 50 international unit/mL penicillin, and 50 mg/mL streptomycin. The DPSCs were maintained in T-25 cm^2^ culture flasks under standard incubation conditions (37°C, 95% air/5% CO_2_) until they reached approximately 70%-80% confluence. After one week of confluence, cells were fragmented with trypsin solution and seeded onto six-well plates with a density of 2.5 x 10^5^ cells per well. The wells were supplemented with 2 mL of complete growth medium [21].

MTT assay

To conduct the 3-[4,5-dimethylthiazol-2-yl]-2,5 diphenyltetrazolium bromide (MTT) assay, 1 mL of culture medium was introduced into each well of the six-well plate. Then, 0.5 mg/mL of MTT was applied to the bottom well. The plate was subsequently placed in an incubator for a duration of four hours at 37°C. After completion of the incubation period, the culture medium was removed from both the insert and the well. The resulting formazan crystals were then dissolved by adding 100 µL of dimethyl sulfoxide (DMSO) solution per well. Gentle shaking for two minutes ensured that the blue reaction product was thoroughly mixed with the solvent. Following this step, 100 µL of the colored DMSO solution from each insert and well was transferred to a new 96-well plate to quantify cell viability. Finally, the absorbance at 450 nm was determined using a microplate reader [22].

Statistical analysis

The findings were expressed as the mean of triplicates accompanied by their respective standard deviations. Statistical analysis was performed using the GraphPad Prism software version 5.03 (GraphPad Software Inc., La Jolla, CA). A one-way analysis of variance was employed to assess the significance level between the control and other experimental groups.

## Results

Developmental toxicity in embryos

**Figure 1:**
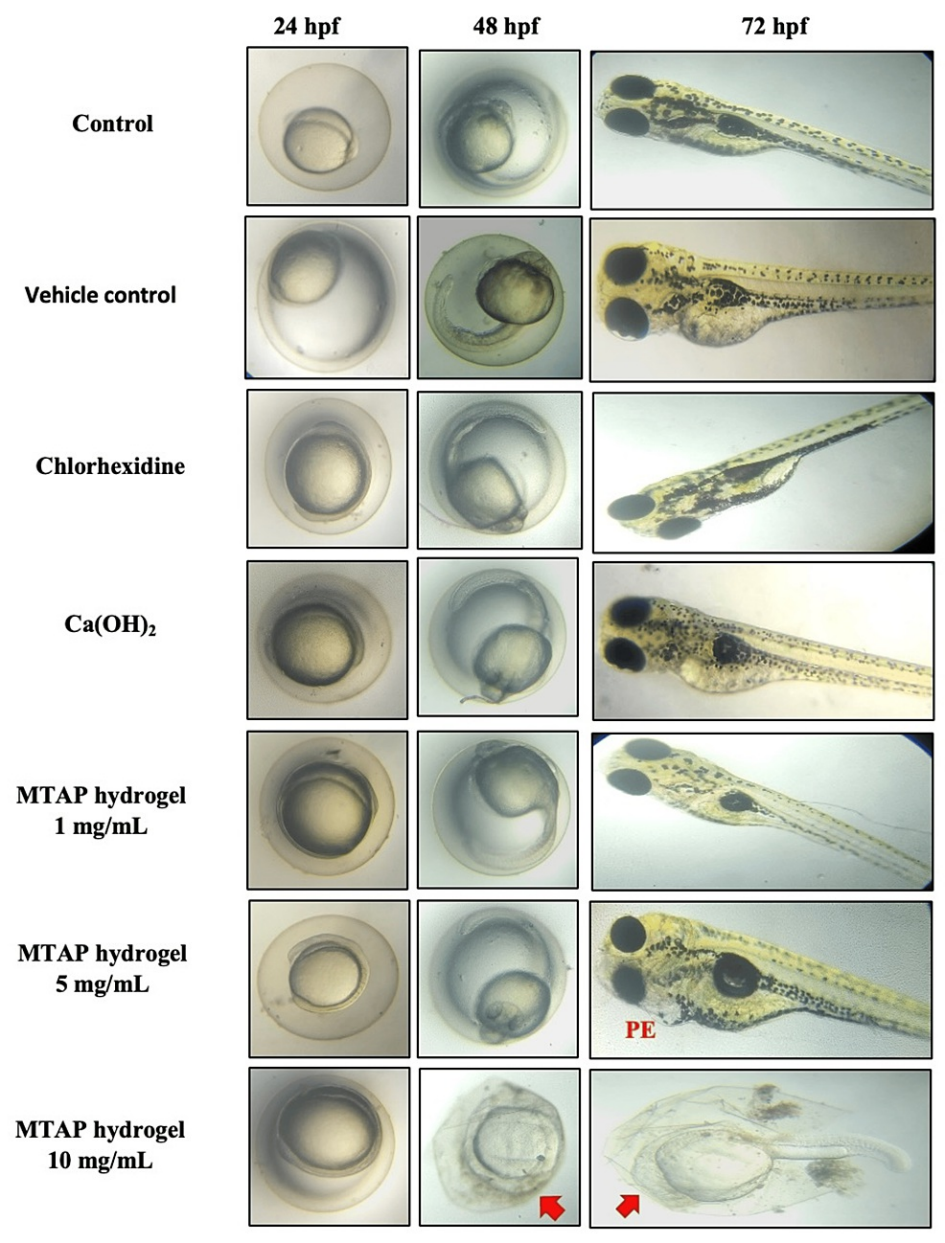
Developmental toxicity analysis was investigated in the zebrafish embryos/larvae from 24 to 72 hpf. Red arrows indicate the developmental toxicity condition hpf: hours postfertilization; Ca(OH)2: calcium hydroxide; MTAP: modified triple antibiotic hydrogel; PE: pericardial edema

The obtained results indicate the absence of developmental malformations in the control group, CHX group, calcium hydroxide group, and MTAP at 1 mg/mL. However, notable malformations among the zebrafish are observed at concentrations of 5 mg/mL, characterized by pericardial edema, and at 10 mg/mL of MTAP, characterized by challenges in transitioning from the embryo stage to larvae development (Figure 1).

Heart rate in zebrafish embryos

**Figure 2:**
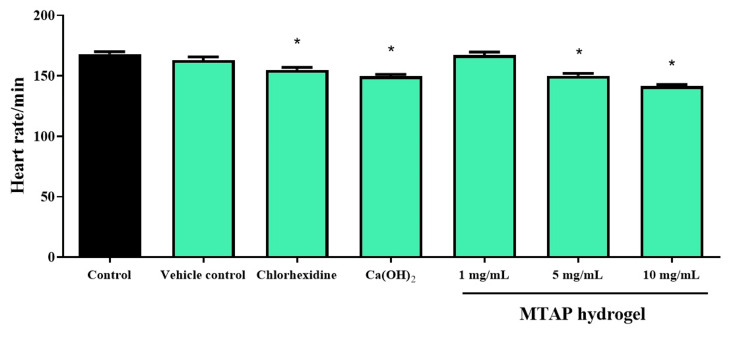
The heart rate of zebrafish embryos was investigated after being exposed to different groups for 24 hours *Significant difference between the control (untreated embryos) and treated groups (F value = 14.2; p < 0.05) Ca(OH)2: calcium hydroxide; MTAP: modified triple antibiotic hydrogel

The embryonic heart rate was recorded for one minute under the microscope, and the average heart rate per minute was subsequently documented. The application of MTAP hydrogel at concentrations of 1 mg/mL and the vehicle control did not significantly impact the heart rates of zebrafish embryos. Conversely, a substantial drop in heart rate was seen when embryos were subjected to treatments involving standard medicaments such as calcium hydroxide and CHX. Elevated concentrations of MTAP hydrogel at 5 and 10 mg/mL resulted in observable changes in heart rates, as illustrated in Figure 2.

Hatching rate in zebrafish embryos

**Figure 3:**
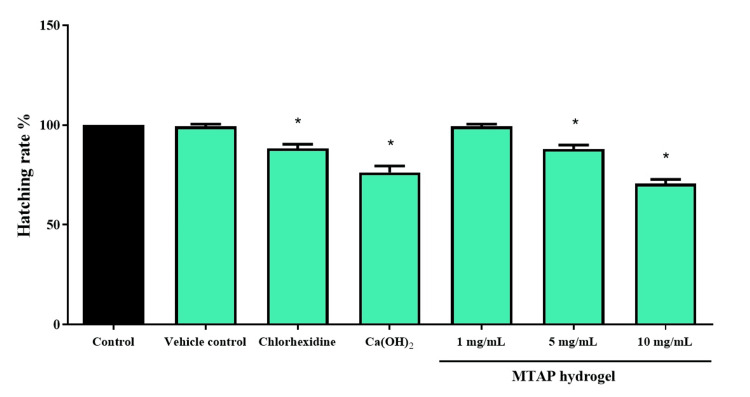
The hatching rate of zebrafish embryos was investigated after being exposed to different groups for 24 hours *Significant difference between the control (untreated embryos) and treated groups (F value = 16.5; p < 0.05) Ca(OH)2: calcium hydroxide; MTAP: modified triple antibiotic hydrogel

Assessing hatching rates in zebrafish embryos serves as a crucial measure to evaluate the potential effects of various interventions. Notably, while calcium hydroxide and CHX exhibited a significant reduction in hatching rates, MTAP hydrogel only displayed distinct changes at higher concentrations, particularly at 5 and 10 mg/mL, indicating alterations in developmental outcomes compared to these chemical-based medicaments, as depicted in Figure 3. Embryos treated with MTAP hydrogel at concentrations of 1 mg/mL and plain carrageenan (vehicle control) showed no significant impact on the hatching rates of zebrafish embryos.

Survival rate in zebrafish embryos

**Figure 4:**
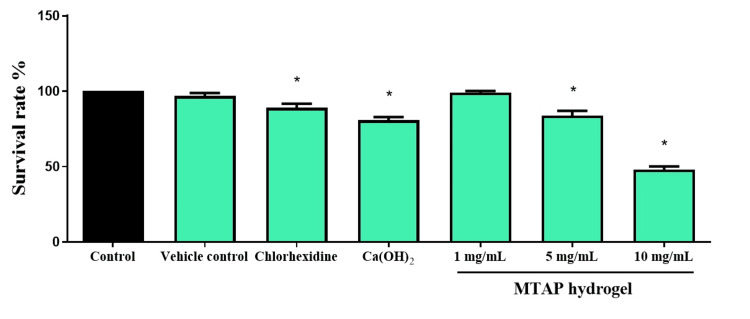
The survival rate of zebrafish embryos was investigated after being exposed to different groups for 24 hours *Significant difference between the control (untreated embryos) and treated groups (F value = 13.3; p < 0.05) Ca(OH)2: calcium hydroxide; MTAP: modified triple antibiotic hydrogel

The survival rate analysis in zebrafish embryos yielded intriguing findings regarding the effects of various substances on their viability. Notably, while the CHX and calcium hydroxide groups showed decreased survival rates, concentrations of 5 and 10 mg/mL of MTAP hydrogel also exhibited reduced survival rates. In contrast, the control group and the MTAP at 1 mg/mL showed no statistically significant differences across the corresponding parameters. These results suggest potential drawbacks associated with calcium hydroxide and CHX compared to MTAP hydrogel, highlighting the comparatively higher survival rates of MTAP hydrogel, especially noticeable at the 1 mg/mL concentration (Figure 4).

Neutral red assay in zebrafish embryos

**Figure 5:**
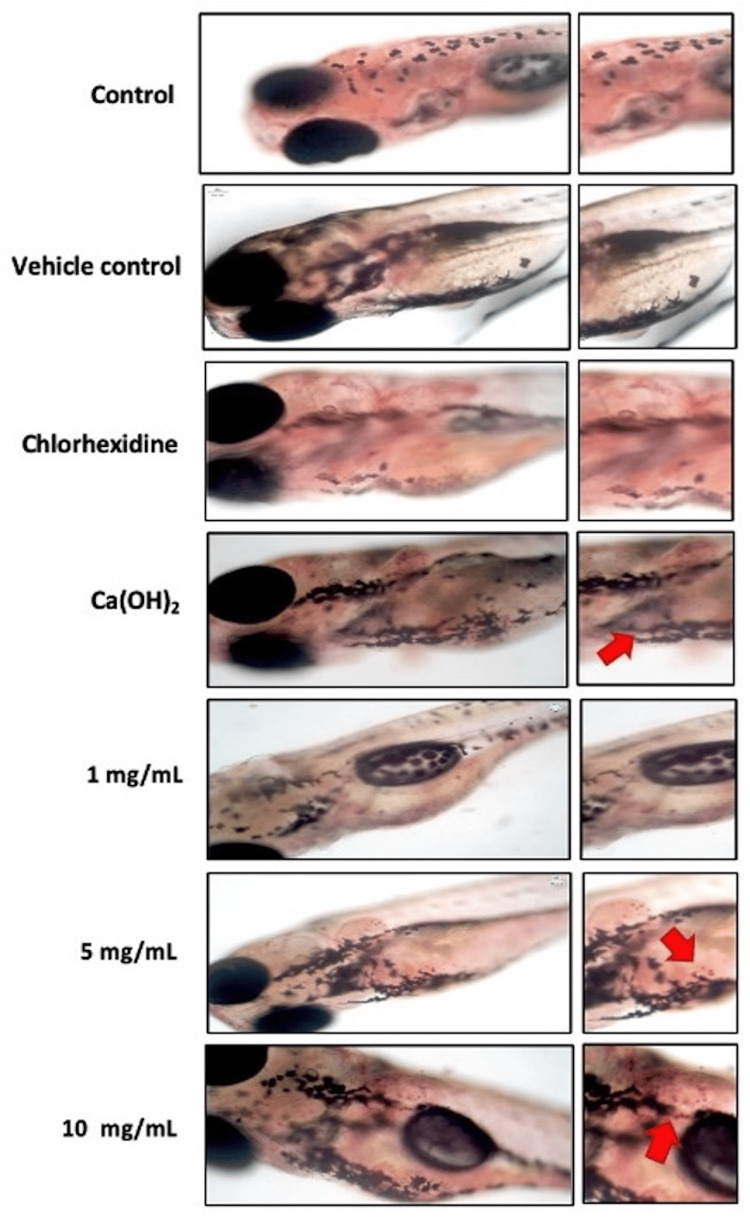
The macrophage migration in larvae due to toxicity conditions was observed through neutral red staining. Red arrows show the macrophages in larvae Ca(OH)2: calcium hydroxide

**Figure 6:**
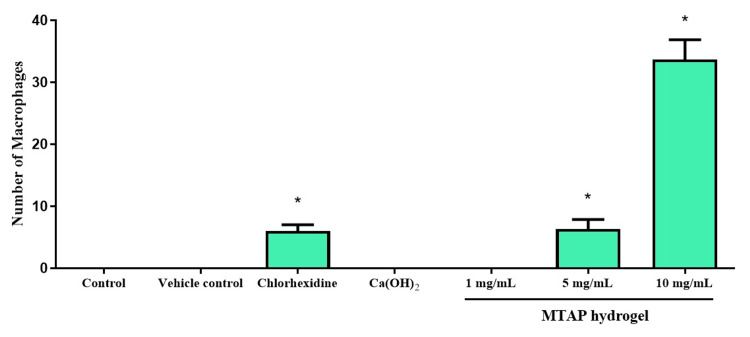
The number of macrophages in larvae is presented in graphs *Significant difference between the control and treatment groups (F value = 16.8; p < 0.05) Ca(OH)2: calcium hydroxide; MTAP: modified triple antibiotic hydrogel

The experimental groups included the control, CHX, CaOH_2_, 1, 5, and 10 mg/mL of MTAP hydrogel. The neutral red assay revealed the migration of macrophages, visible as red dots indicating inflammation, in the zebrafish exposed to these medicaments, as depicted in Figure 5. Significant toxicity was observed in the CHX group and in the modified triple antibiotic hydrogel at concentrations of 5 and 10 mg/mL, as illustrated in Figure 6, when compared to other groups.

MTT assay

**Figure 7:**
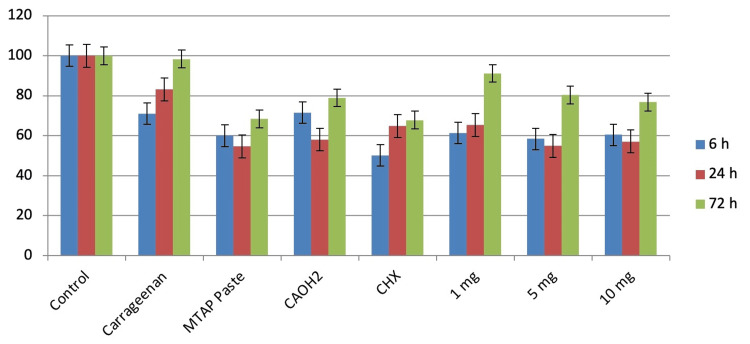
Cytotoxicity assessment at 6, 24, and 72 hours CaOH2: calcium hydroxide; MTAP: modified triple antibiotic hydrogel; CHX: chlorhexidine

The cytotoxicity of the medicaments exhibited variability across various concentrations and time intervals. The experiments were conducted in triplicate. The evaluation of cytotoxicity against human dental pulp stem cells (hDPSCs) is portrayed in a manner dependent on both dosage and duration, encompassing timeframes of 6, 24, and 72 hours, and the results are expressed in mean (M). Figure 7 illustrates the cell viability percentages for various root canal medicaments at different concentrations at 6, 24, and 72 hours. The study's results significantly impact hDPSCs with higher concentrations of modified triple antibiotic hydrogel, CaOH_2_, and CHX.

## Discussion

ICMs are commonly administered during the intervals between appointments, offering several benefits. They function by impeding the proliferation of bacteria and reducing the infiltration of pathogens during these intervals, often occurring through a compromised temporary restoration [23]. The choice of the most suitable ICM for eradicating root canal infection before commencing endodontic procedures or regenerative procedures should be guided by two primary objectives: first, the optimization of antibacterial efficacy, and second, the preservation of the proliferative and differentiative capacities inherent to pulp stem cells. Recent investigations have illuminated that the concentrations of intracanal medications commonly employed in endodontic procedures exert detrimental impacts on SCAP and DPSCs [24,25]. Consequently, this study endeavors to address this concern by formulating a modified triple antibiotic hydrogel at concentrations spanning 1, 5, and 10 mg/mL, with the intention of conducting a thorough evaluation of its potential cytotoxicity.

Cytotoxicity is defined as the ability of a substance to influence cellular viability. This phenomenon can be assessed through diverse physiological indicators, encompassing diminished cell growth and proliferation, occurrences of necrosis and apoptosis, or a convergence of these factors [26]. In this study, cytotoxicity was assessed using both the MTT assay and a zebrafish larvae model. This dual approach was employed because a single cytotoxicity test cannot comprehensively evaluate or predict all aspects of a material's cytotoxicity. 

Investigating developmental toxicity in zebrafish embryos provides a vital method for gaining essential insights into potential risks to human health and the environment [15]. Furthermore, it acts as a mechanism for uncovering the underlying pathways responsible for the toxicity of these agents, thereby aiding in the development of new strategies aimed at alleviating or preventing their adverse effects. In the current investigation, it was observed that with the increase in the concentration of the antibiotic hydrogel, there was a discernible increase in the manifestation of developmental malformations.

Exploring heart rate dynamics in zebrafish embryos serves as a valuable means for understanding the mechanisms governing heart development and function. Additionally, this analysis has the potential to identify prospective drug targets for treatment strategies [27]. Analyzing hatching rates in zebrafish embryos provides crucial insights for both basic and applied dental research. This includes studying the detailed mechanisms of embryo development and the effects of environmental stressors on embryo health [18]. Examining heart rate, hatching rate, and survival rates in zebrafish embryos provides crucial insights for both basic and practical research. Within the framework of this study, it became evident that commercially available ICMs indeed influence the heart rate, survival rate, and hatching rate of zebrafish, thereby establishing a state of cytotoxicity. The examination also unveiled that elevated concentrations of antibiotics were associated with an adverse impact on zebrafish survival and DPSC survival. Nonetheless, a concentration of 1 mg/mL for the modified triple antibiotic hydrogel showed no discernible effect on the survival rate of zebrafish. A study by Saberi et al. assessed the cytotoxic efficacy of medicaments on SCAP and found that CaOH_2_ exhibited the highest cytotoxicity, while lower concentrations of MTAP demonstrated better cytocompatibility [28]. These results align with the findings obtained in this study, demonstrating a consistent pattern. Another study by Jamshidi et al. [29] concluded that antibiotic pastes, particularly at higher concentrations, induced cytotoxicity and genotoxicity in human SCAP. This study highlights an inverse relationship between the concentration of antibiotic pastes and the viability of these stem cells, emphasizing the need for careful consideration of dosage in clinical applications to minimize harmful effects on cell health.

The utilization of the neutral red assay in zebrafish larvae is widely applied in both basic and advanced dental research, facilitating investigations into immune responses triggered by infections and the impact of toxins on immune function [20]. It is a potent instrument for exploring macrophage function within zebrafish larvae, offering extensive utility across a spectrum of fundamental and practical research pursuits. Within the context of this investigation, notable toxicity was observed in the CHX group and at 5 and 10 mg/mL of antibiotic hydrogel. Upon staining, these specific groups displayed indications of toxicity, manifested as red dots suggestive of macrophage presence. These findings are similar to a study carried out by Ruparel et al., which underscores that elevated antibiotic concentrations adversely impact the viability of SCAP, while lower concentrations are conducive to SCAP survival and proliferation [9]. Additionally, research conducted by Faria et al. emphasizes that pastes formulated with a polymer medium vehicle exhibit diminished cytotoxicity compared to pastes containing water [30]. Consequently, innovative carrier mediums utilizing biomaterials have been developed, demonstrating sustained drug-release properties.

The limitations of this study include the narrow focus on specific concentrations or application methods of MTAP hydrogel. Additionally, there is a lack of comprehensive analysis regarding the long-term effects or broader implications of MTAP hydrogel utilization in oral health interventions. Future research efforts should aim to address these limitations by undertaking comprehensive inquiries into MTAP hydrogel, thereby broadening the understanding of this domain. Additional investigations are necessary to refine formulations and explore the clinical viability of MTAP in endodontic and regenerative procedures, emphasizing the importance of careful dosage considerations in dental applications.

## Conclusions

From the results, it is illustrated that a higher concentration of hydrogel showed toxic effects; however, the hydrogel at a lower concentration of 1 mg/mL showed no toxic effect in zebrafish embryos and larvae. These findings highlight the necessity of optimizing ICM concentrations to strike a balance between antibacterial efficacy and minimal cytotoxicity.
